# Bactericidal/Permeability-Increasing Protein (BPI), a Novel Antimicrobial Molecule in Human Breast Milk with Immune Potential

**DOI:** 10.3390/microorganisms13010115

**Published:** 2025-01-08

**Authors:** Alba Soledad Aquino-Domínguez, María de los Ángeles Romero-Tlalolini, Honorio Torres-Aguilar, Juan Carlos Rodríguez-Alba, Lucia Lourdes Martínez-Martínez, Francisco Javier Sánchez-Peña, María Teresa Hernández-Huerta, Jesús Elizarrarás-Rivas, Gabriela Tapia-Pastrana, Melisa Gómez-López, Elsa Cruz-Martínez, Uriel Eleazar Hernández-Corrales, Sergio Roberto Aguilar-Ruiz

**Affiliations:** 1 Facultad de Medicina y Cirugía, Universidad Autónoma “Benito Juárez” de Oaxaca, Oaxaca 68120, Oaxaca, Mexico; asaddominguez@gmail.com (A.S.A.-D.); lumartin1969@yahoo.com (L.L.M.-M.); kowalski-23@hotmail.com (F.J.S.-P.); dr.jesuselizarraras@gmail.com (J.E.-R.); 2 Consejo Nacional de Humanidades, Ciencias y Tecnologías, Facultad de Medicina y Cirugía, Universidad Autónoma “Benito Juárez” de Oaxaca, Oaxaca 68120, Oaxaca, Mexico; romerotlalolini@gmail.com (M.d.l.Á.R.-T.); mthernandez@conahcyt.mx (M.T.H.-H.); 3 Facultad de Ciencias Químicas, Universidad Autónoma “Benito Juárez” de Oaxaca, Oaxaca 68120, Oaxaca, Mexico; qbhonorio@hotmail.com; 4 Unidad de Neuroinmunología y Neurooncología, Instituto Nacional de Neurología y Neurocirugía, Ciudad de México 14269, Mexico; juan.rodriguez@innn.edu.mx; 5 Coordinación de Investigación en Salud, Instituto Mexicano del Seguro Social (IMSS), Oaxaca 68000, Oaxaca, Mexico; 6 Servicios de Salud del Instituto Mexicano del Seguro Social para el Bienestar (IMSS-BIENESTAR), Hospital del Alta Especialidad de Oaxaca, San Bartolo Coyotepec 71294, Oaxaca, Mexico; gabrielatapiapastrana@gmail.com; 7 Hospital General “Aurelio Valdivieso MD”, Secretaria de Salud (SS), Oaxaca 68000, Oaxaca, Mexico; melaza2001@hotmail.com (M.G.-L.); elssa20@hotmail.com (E.C.-M.); 8 Hospital Comunitario Nejapa de Madero, Nejapa de Madero 70531, Oaxaca, Mexico; dr.uriel.gineco@gmail.com

**Keywords:** breast milk, bactericidal/permeability-increasing protein (BPI), colostrum, antimicrobial function, anti-inflammatory function, epithelial cells, leukocytes

## Abstract

Breast milk is a fluid of vital importance during the first stages of life of the newborn since, in addition to providing nutrients, it also contains cells and molecules of the immune system, which protect the neonate from infection and, at the same time, modulate the establishment of the microbiota. Bactericidal/permeability-increasing protein (BPI) is relevant in preventing disease and sepsis in neonates. Therefore, the following work aimed to demonstrate the presence of BPI in the different stages of breast milk and its possible immune functions. Our results demonstrate for the first time the presence of soluble BPI and leukocytes and epithelial cells containing it, primarily in the colostrum stage. Using BPI at concentrations typical of colostrum, we observed that it reduces the growth of two distinct *E. coli* strains, enhances the uptake of these bacteria by monocytes, and suppresses the secretion of the proinflammatory cytokine interleukin (IL)-8 in infected intestinal cells. These findings suggest that BPI transferred via colostrum from mother to newborn may play a significant role in providing antimicrobial and anti-inflammatory protection during the early stages of life.

## 1. Introduction

In the critical phase of developing their immune system, newborns are exceptionally susceptible to infections. This vulnerability underscores the crucial role of antimicrobial proteins and peptides (APPs) such as defensins, cathelicidins, lactoferrins (LFs), lysozymes, and bactericidal/permeability-increasing proteins (BPIs), among others, in their protection. However, during the initial months of life, particularly in premature or low-birth-weight babies, the levels of these APPs are typically low, heightening the risk of infections. In these circumstances, the role of APPs in combating pathogenic microorganisms and controlling inflammation becomes even more vital. Several studies have demonstrated that administering exogenous APPs can significantly reduce the risk of infections in neonates and infants [1]. Breast milk, a rich exogenous source of APPs, provides additional protection to the newborn [2,3].

Antimicrobial peptides (AMPs) are characterized by a net positive charge (+2 to +13). The cationic nature can be attributed to the presence of lysine, arginine, and histidine residues. In addition, AMPs are hydrophobic. These structural and physicochemical characteristics allow AMPs to bind electrostatically to the outer face of Gram-negative and Gram-positive bacteria membranes, culminating in membrane damage in a manner dependent or independent of pore formation. AMPs can inhibit both cell wall synthesis and bacterial proteins and nucleic acids [4]. Lactoferrins (LFs), one of the most abundant proteins in breast milk or its derived peptides, inhibit the growth of different bacteria by mechanisms involving iron sequestration or direct interaction with the bacterial membrane, which is a similar mechanism employed by AMPs [5,6]. Moreover, APPs have a variety of immune functions, including activation, attraction, and differentiation of leukocytes, stimulation of angiogenesis, and reduction in proinflammatory cytokine production and reactive oxygen species. Some APPs can neutralize Gram-negative bacterial toxins and lipopolysaccharides (LPSs) and prevent interaction with its receptor, the Toll-like receptor (TLR)-4, which is expressed in different cell types since the LPS-TLR4 interaction triggers a response that, in some cases, can become uncontrolled, producing inflammation and organ damage [4,7], hallmarks of neonatal sepsis, which is one of the leading causes of mortality in neonatal intensive care units (ICUs) [8,9].

Bactericidal/permeability-increasing protein (BPI), a cationic molecule that shares antimicrobial functions with breast milk molecules, but which has not been reported in this biological fluid, also alters the membranes of microorganisms directly. In addition, they bind to microbes to mark them for destruction by phagocytic cells through an opsonization mechanism. In addition, they perform immunomodulatory functions, such as regulating inflammation, because they neutralize the LPSs of Gram-negative bacteria, which is crucial during infections to combat them without generating an inflammatory process. BPIs have a remarkable antimicrobial and immunomodulatory capacity, especially due to their affinity for LPSs [10].

Its deficiency in neonates is associated with an increased risk of Gram-negative bacterial infections, and sepsis is significant in protecting premature neonates. A study in the United Kingdom and the United States demonstrated that infants with severe meningococcal sepsis who received intravenous recombinant BPI (rBPI21) or albumin solution as a control had less involvement (3.2% vs. 7.4%) and better functional outcomes demonstrating the potential benefit of IFS in protecting against severe infections in newborns [11]. The potential benefit of BPI reinforces its importance in the fight against severe infections in newborns. Finally, considering the importance of antimicrobial molecules in breast milk and without the intention of displacing breastfeeding by artificial feeding, studies such as the multicenter ELFIN UK trial with 2200 infants demonstrate that exogenous lactoferrin in fortified formulas helps fight infections in severely ill and deficient-birth-weight infants. Emphasizing the importance of supplementing milk formulas with antimicrobial molecules present in breast milk in neonates or infants without access to breastfeeding [12]. In addition, BPI-deficient murine models that have experimentally induced colitis suffer greater affectations than wild-type mice [13]. Therefore, the following article aimed to identify the presence of BPI in breast milk, the cells that produce it, and its antibacterial, opsonizing, and anti-inflammatory potential.

## 2. Materials and Methods

### 2.1. Sample Collection

Samples were collected from two state hospitals. The teaching, training, and research Committee of the General Hospital “Dr. Aurelio Valdivieso” approved this study. Additionally, the General Hospital of the Mexican Institute of Social Security (IMSS), Zone 1 in Oaxaca, granted its approval under authorization number R-2023-2001-023. The women gave their data and consent before starting any study procedure or sample collection in full compliance with the ethical guidelines. Inclusion criteria were being clinically healthy, having a maternal age of 18 years, and having the intention to breastfeed. Seventy-five participants were recruited. The collection of breast milk (BM) from the participants was a meticulous process, conducted at three time points to represent each stage: colostrum (C; day 2–5; 1–2 mL), transition milk (TM; day 8–12; 3–5 mL), and mature milk (MM; day 26–30; 5–10 mL). This process, guided by the methodology of Trend et al. [14], ensured the reliability of the samples. Participants were provided with detailed instructions on hygienic milk collection, such as hand and nipple asepsis with soap and water before milk collection, and sterile containers were used. The milk was extracted manually, according to the donor’s usual method or as instructed by the staff, and an aliquot was extracted with a sterile Pasteur pipette into a 15 mL plastic tube. The whole BM was stored at 4 °C until collected by research personnel and transported to the research laboratory on ice. The volume of milk donated to the study was recorded at each visit before processing.

### 2.2. Quantification of BPI in Breast Milk

The presence of BPI was quantified in the protein fraction or serum of the milk by an enzyme-linked immunosorbent assay by the human BPI ELISA (DuoSet^®^ ELISA Development System (DY7468-05), R&D Systems, Minnneapolis, MN, USA) following the manufacturer’s recommendations as described. To a plate coated with 100 μL of the detection monoclonal antibody (mAb) previously incubated overnight, 100 μL of the samples was added and incubated at room temperature for 2 h. Immediately, the plate was aspirated and washed with 400 μL of wash buffer. Subsequently, the detection antibody was added, allowing its interaction with the sample by incubating for 2 h at room temperature. After this period, the plate was rewashed, and the streptavidin–HRP enzyme solution was added, leaving it to incubate for 20 min at room temperature and protected from light. The plate was washed, and then the enzyme substrate solution was added, allowing its interaction during a 20-min incubation at room temperature, protected from light, and finally, 50 μL of the stop solution was added to the enzymatic reaction. The concentration of the sample was determined by measuring the optical absorbance at 450 nm using a ChroMate plate reader (Awareness Technology, Palm City, FL, USA). The absorbance value was then used in a linear calibration equation to calculate the sample concentration, ensuring precise quantification. During the quantification of the sample, dilution curves were performed to ensure that each of the analyzed samples fell within the detection range of the ELISA assay. ELISA tests were conducted in groups of samples grouped by different dilutions. Each sample was diluted and analyzed in duplicate. The colostrum group required the highest dilution, followed by the transition milk group, and finally, most mature milk samples did not require dilution. Afterward, the results were multiplied by their dilution factor and graphed.

### 2.3. Identification of BPI-Producing Cell Populations in Breast Milk

Cells recovered from colostrum and mature milk samples were incubated in 100 µL and suspended in a Cytofix/Cytoperm (BD Biosciences, Franklin Lakes, NJ, USA) flow cytometry permeabilization buffer at approximately 1 × 10^6^ cells/mL for 30 min. Afterward, once the cells were permeabilized, they were incubated for 30 min with the following mAbs at room temperature: anti-human BPI Alexa Fluor 488 or the anti-IgG Alexa Fluor 488 isotype control (R&D Systems, Minnneapolis, MN, USA). To identify leukocyte lineage, surface staining was performed with the APC mouse anti-human CD11b/Mac-1 or APC mouse IgG1 κ isotype control (BD Pharmingen, Franklin Lakes, NJ, USA). Lineage identification of epithelial cells was performed with the Alexa Fluor 647 mouse anti-human cytokeratin-14, -15, -16, and -19 conjugated antibody or its corresponding Alexa Fluor 647 mouse anti-human isotype control (Becton, Dickinson, Franklin Lakes, NJ, USA). After staining, breast milk cells or blood leukocytes were washed, and excess unbound antibodies were removed to resuspend in 300 μL of stabilizing fixative. The fixed samples were processed with the MACSQant cytometer (Milteny Biotec, Bergisch Gladbach, Germany), and the images were analyzed with FlowJo V10 software (FlowJo, Ashland, OR, USA).

### 2.4. Cell Lines and Bacterial Strains

The human colorectal adenocarcinoma cell line HT-29 (ATCC HTB-38) was kept alive and proliferating in a high-glucose DMEM culture medium (Biowest, Nuaillé, France) supplemented with 0.1 mM stable glutamine, 100 U penicillin/mL, 100 μg streptomycin/mL, and 10% FBS (fetal bovine serum) (Biowest, Nuaillé, France). The human acute monocytic leukemia cell line THP-1 (ATCC TIB-202) was cultured in an RPMI 1640 medium (Merck KGaA, Darmstadt, Germany) with 100 U penicillin/mL, 100 μg streptomycin/mL, and 10% FBS. All cell lines were maintained at 37 °C in an incubator (ECOSHEL 9052, Fort Worth, TX, USA) with 5% CO_2_.

The reference strains of *E. coli* strain Seattle 1946 (ATCC 25922) and the clinical isolate used in this study were kindly donated by Dr. Martinez-Martinez Lucia Lourdes from the School of Faculty of Medicine and Surgery of the Autonomous University “Benito Juárez of Oaxaca”. The clinical isolate was obtained from a urine sample of a pediatric patient diagnosed with a urinary tract infection at the Children’s Hospital of Oaxaca. The isolate was identified and characterized using standard clinical microbiology techniques, including biochemical testing, partial sequencing of the 16S rRNA gene, and antibiogram analysis. The clinical isolate was resistant to ampicillin and trimethoprim/sulfamethoxazole. However, it remained susceptible to a broad spectrum of antibiotics, including amikacin, cephalothin, cefepime, cefotaxime, ceftazidime, ceftriaxone, cefuroxime, ciprofloxacin, ertapenem, fosfomycin, gentamicin, levofloxacin, linezolid, meropenem, moxifloxacin, nitrofurantoin, norfloxacin, tetracycline, tigecycline, and vancomycin. The strain tested negative for extended-spectrum β-lactamase (ESBL) production. However, no data were available on hypervirulence or hyperviscosity phenotypes. Both strains were preserved at −70 °C in lysogeny broth (LB) containing 10% glycerol. For experimental procedures, bacterial cultures were revived in LB and incubated overnight at 37 °C with shaking at 80 rpm. Before use, overnight cultures were transferred to a DMEM medium without fetal bovine serum (FBS) or antibiotics and incubated for one hour at 37 °C without shaking.

### 2.5. Antimicrobial Activity Assays

The bacterial viability assay was performed by a standardized methodology using the colorimetric reagent resazurin sodium salt (Merck, Rahway, NJ, USA, EE.UU.) with a count of 150,000 CFU/mL of *E. coli* strain Seattle 1946 and a clinical isolate, using a 96-well plate, where 170 µL of Mueller–Hinton broth and 30 µL of 0.02% resazurin were placed, and in some wells, the treatment with recombinant BPI (His Tag) (Sino Biological, Chesterbrook, PA, USA) was added at a concentration of 1.645, 0.236 µg/mL, or 0.638 ng/mL; as a negative control, 5 µL of amikacin 1.25 mg/mL was used. The plates were placed in the multimode reader through the Multiskan™ FC Microplate Photometer (Thermo Scientific, Waltham, MA, USA). The absorbance measurement was performed at 620 nm (resazurin) and 570 nm (resorufin) at times of 0, 6, 6.5, 7.0, 8.0, and 24 h. In other experiments, milk formulas (Frisolac GOLD, The Hague, The Netherlands) were supplemented with recombinant BPI, using the concentration of 1645 µg/mL to evaluate the viability of *E. coli* strains according to the methodology described above.

### 2.6. E. coli Uptake Assays by the THP-1 Cell Line

A total of 1.5 × 10^6^ CFU of the reference strain *E. coli* Seattle 1946 or the clinical isolate of *E. coli* was stained with 10 µM CFSE for 15 min, shaken at 80 rpm, and protected from light, and then two washes with PBS removed the excess dye. Later, they were subjected to one of the treatments without or in the presence of 1.645 μg/mL of recombinant BPI or human plasma for 10 min. Stained and treated bacteria were in contact with 1 × 10^5^ THP-1 cells for one minute at 37 °C shaking at 80 rpm. Finally, THP-1 cells were fixed with 4% paraformaldehyde for 30 min and acquired on a MACSQuant flow cytometer (Miltenyi Biotec, Cologne, Germany). The data obtained were analyzed using FlowJo Version 10 software.

### 2.7. Interaction of BPI in Colostrum with E. coli

The total protein fraction was recovered from human colostrum samples by centrifugation at 10,000× *g*/15 min at 4 °C; then, 200 µL of the protein concentrate was used to suspend a concentration of 1 × 10^6^ CFU of the reference strain *E. coli* Seattle 1946 and to allow their interaction for 20 min under 80 rpm agitation at 4 °C. BPI binding to the bacteria was then detected using the anti-human BPI or its respective isotype control (Section 2.3). Finally, the bacteria were fixed and analyzed on a flow cytometer, and the images were processed with FlowJo software.

### 2.8. Secretion of IL-8 by HT-29 Cells

Using an enzyme-linked immunosorbent assay, ELISA, for human IL-8/CXCL8 (DuoSet and Systems, Minneapolis, MN, USA) according to the manufacturer’s specifications, the concentration of the proinflammatory cytokine IL-8 was measured in the culture supernatant of 3 × 10^5^ intestinal epithelial cells of the HT-29 line (ATCC^®^-CRL-2021™, Manassas, VA, USA) with 1.5 × 10^5^ CFU of the *E. coli* Seattle 1946 strain or the clinical isolate (Section 2.4) after two hours of interaction being additionally treated with medium concentrations of recombinant BPI (Section 2.2) during the lactation phases colostrum (1.645 µg/mL), transition (0.638 µg/mL), mature milk (0.638 ng/mL), or amikacin 1.25 mg/mL.

## 3. Results

### 3.1. Human Breast Milk Contains BPI

**Figure 1:**
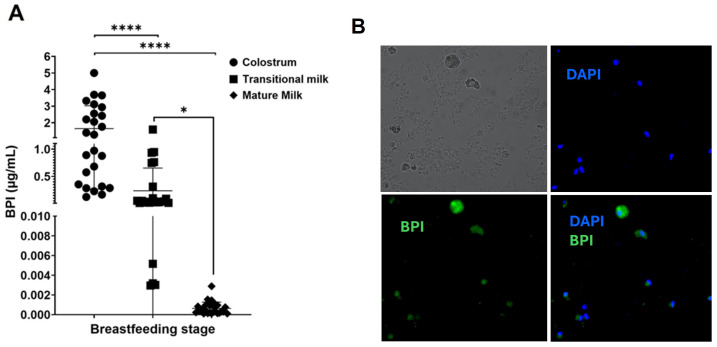
Human breast milk contains BPI. (**A**) Human breast milk samples were collected from 75 clinically healthy lactating women, with 25 samples per stage: colostrum (1–5 days postpartum), transitional milk (6–15 days postpartum), and mature milk (16 days to 6 months postpartum). BPI levels were measured by ELISA. The graph shows the concentration obtained in each of the milk samples per individual and the average and standard deviation per stage. Statistical analysis was performed by Tukey’s multiple comparison test, with significance indicated as follows: * *p* < 0.05, and **** *p* < 0.0001. (**B**) Cells were purified from colostrum samples from clinically healthy women (2 days postpartum), fixed on slides, permeabilized, and stained with a monoclonal antibody (mAb) against BPI. Nuclei were counterstained with DAPI to identify cells. Representative images are shown from one of three independent assays in which five fields were analyzed per sample. Brightfield image of colostrum cells (**top left**), staining with DAPI (blue) to highlight nuclei (**top right**), immunofluorescence staining for BPI (green) (**bottom left**), and the combined image showing the colocalization of BPI with DAPI (**bottom right**).

**Figure 2:**
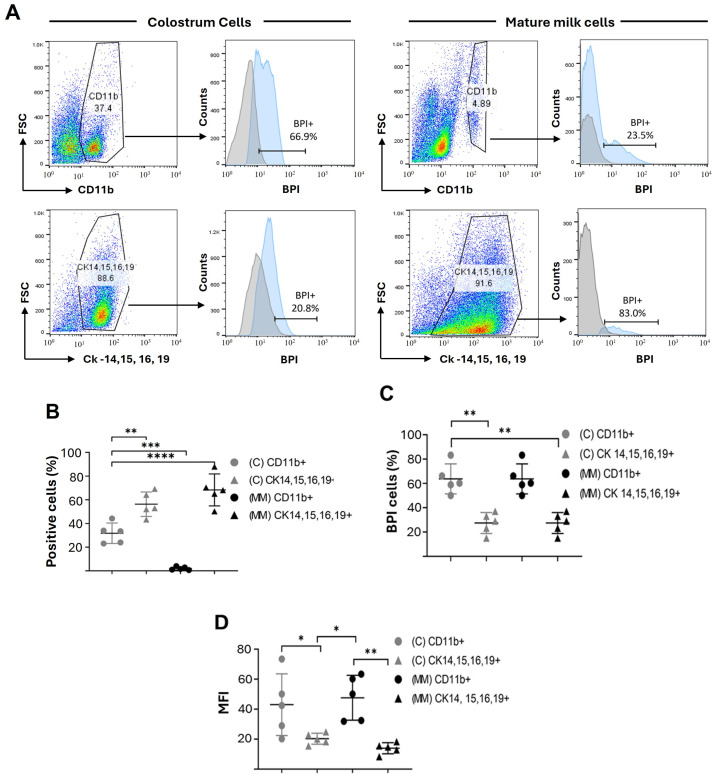
Phenotypic characterization and intracellular expression of BPI in colostrum and mature milk cells. (**A**) Flow cytometry analysis of cells from colostrum and mature milk shows populations expressing CD11b (a myeloid cell marker) and cytokeratins CK14, CK15, CK16, and CK19 (epithelial cell markers). The forward scatter (FSC; x-axis) plots display gated populations, indicating the percentage of positive cells. Corresponding histograms represent intracellular BPI expression in these gated populations (blue) compared to isotype controls (gray). The x-axis of the histograms denotes fluorescence intensity, while the y-axis indicates the number of cells (counts). (**B**–**D**) Quantitative data are derived from panel (**A**). Panel (**B**) compares the percentages of CD11b^+^ and CKs^+^ cells between colostrum (**C**) and mature milk (MM). Panel (**C**) shows the percentages of BPI^+^ cells within the CD11b^+^ and CKs^+^ populations in both conditions. Panel (**D**) presents the fluorescence intensity mean (MFI) of intracellular BPI expression in these cell subsets. Data are represented as mean ± SD, and statistical analysis was performed by Tukey’s multiple comparison test, with significance indicated as follows * *p* < 0.05, ** *p* < 0.01, *** *p* < 0.001, and **** *p* < 0.0001 from six independent assays at each stage.

Our first objective was to determine the presence of BPI in breast milk, for which this fluid was collected at the colostrum, transition, and mature stages. Samples were obtained from clinically healthy donors, and BPI concentrations were measured using an BPI ELISA (DuoSet^®^ ELISA Development System (DY7468-05), R&D Systems, Minnneapolis, MN, USA). The results show that colostrum has the highest BPI concentrations (1.645 µg/mL, range: 0.124–5 µg/mL) compared to transitional milk (0.326 µg/mL, range: 0.0296–1.589 µg/mL) and mature milk (0.638 ng/mL, range: 0.10–2.88 ng/mL) (Figure 1A). In addition, immunohistochemistry identified the presence of BPI-containing cells in colostrum (Figure 2B). These results show, for the first time, the presence of soluble BPI in all three stages of breast milk, reaching its highest concentrations in colostrum, and in this same stage of mature milk, we identified BPI^+^ cells.

### 3.2. Leukocytes and Epithelial Cells, Two Crucial Components of Breast Milk, Are Found to Contain BPI

Once we identified the presence of BPI^+^ cells, and based on the background that reports that both leukocytes and epithelial cells are abundant in breast milk [14], we decided to identify which cell population produces BPI in colostrum and mature milk. To achieve this goal, we purified cells obtained from colostrum and mature milk, which were permeabilized and stained with mAbs directed against BPI, CD11b (leukocyte lineage marker), and a cocktail of mAbs directed against cytokeratin (CK)-14, -15, -16, and -19, to identify the lineage of the epithelial cells. Our results show a higher percentage of epithelial cells both in colostrum (63.32 ± 10.34% epithelial cells vs. 31.76 ± 8.63% CD11b^+^ cells) and mainly in mature milk (78.3 ± 13.48% epithelial cells vs. 2.24 ± 1.39% leukocytes) (Figure 2A,B). However, CD11b^+^ BPI^+^ cells are present in a higher percentage in colostrum (65.48 ± 9.45% CD11b^+^BPI^+^ vs. 36.44 ± 9.7% BPI^+^ epithelial cells) and have a higher BPI content (4.2 ± 2.05 MFI of CD11b^+^BPI^+^ cells vs. 2.03 ± 0.36 MFI of epithelial cells BPI^+^). This shows that epithelial cells are more abundant in colostrum and mainly in mature milk than leukocytes. However, leukocytes are the cells with the highest BPI content in both stages of breast milk.

### 3.3. BPI at Average Concentrations Present in Colostrum Can Inhibit the Growth of E. coli

**Figure 3:**
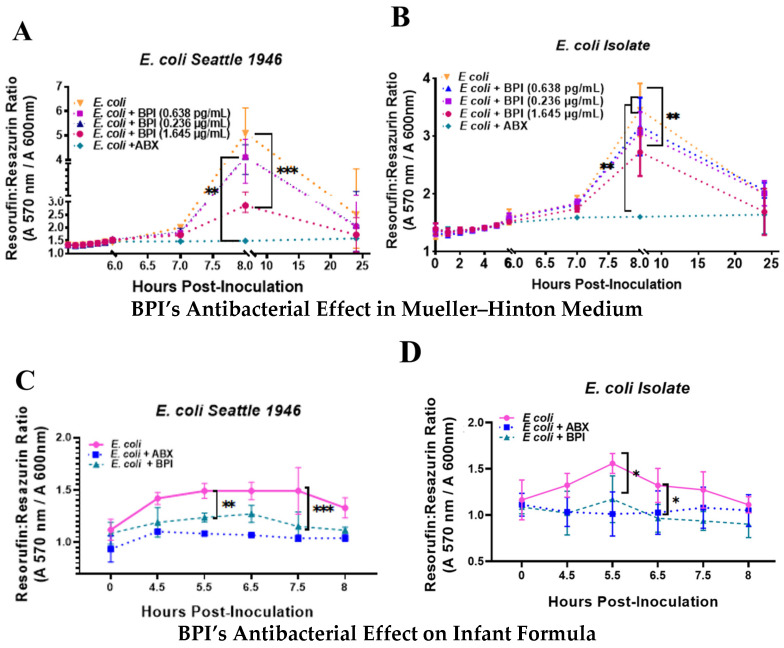
Antibacterial activity of BPI at lactation-relevant concentrations and in BPI-supplemented milk formula. The reference strain *E. coli* Seattle 1946 and (**A**) a clinical isolate of *E. coli* (**B**) were inoculated at 1.5 × 10^3^ CFU/mL and treated with BPI at three concentrations (0.638 ng/mL, 0.236 μg/mL, and 1.645 μg/mL) or amikacin (1.25 mg/mL, positive control) to evaluate bacterial growth inhibition in Muller–Hinton broth over 24 h using a resazurin-based colorimetric assay). Short-term effects (8 h) of BPI (1.645 μg/mL) and amikacin on the reference strain (**C**) or clinical isolate of *E. coli* in the milk formula (**D**). The x-axis represents time post-inoculation (hours), and the y-axis corresponds to metabolic activity, measured as the resorufin/resazurin ratio (A570 nm/A600 nm). Resazurin was used at a final concentration of 0.02%. Data are shown as the mean ± standard deviation (SD) from four independent experiments. Statistical significance was calculated using a two-way ANOVA, denoted as * *p* < 0.05, ** *p* < 0.01, and *** *p* < 0.001.

We considered that the average concentrations of BPI found in the different stages of breast milk exceed the reported inhibitory concentration range (0.0625–1200 µg/mL) [15] to inhibit the growth of *E. coli*. Our next objective was to determine whether the average concentrations of BPI in the different stages of breast milk present this antimicrobial activity. For this purpose, we used a recombinant BPI protein and treated two different strains of *E. coli*, starting from an initial concentration of 150 × 10^3^/mL colony forming units (CFUs), because it is a bacterial load above the values reported in infections of infants and adults [16]. We used the resorufin/resazurin ratio as a bacterial growth tool. The results indicate that BPI has antimicrobial activity against a reference strain (Figure 3A) and a clinical isolate (Figure 3B) of E. coli only when used at colostrum concentration but has no activity at the average concentrations of transitional or mature milk. With this information, we supplemented commercial formula milk with the BPI protein at the average concentration of colostrum (1.645 µg/mL). Similarly, we found that formula milk supplemented with BPI inhibits the growth of both strains used (Figure 3C,D).

### 3.4. BPI Used at Colostrum Concentrations Favors the Uptake of E. coli by THP-1 Cells

**Figure 4:**
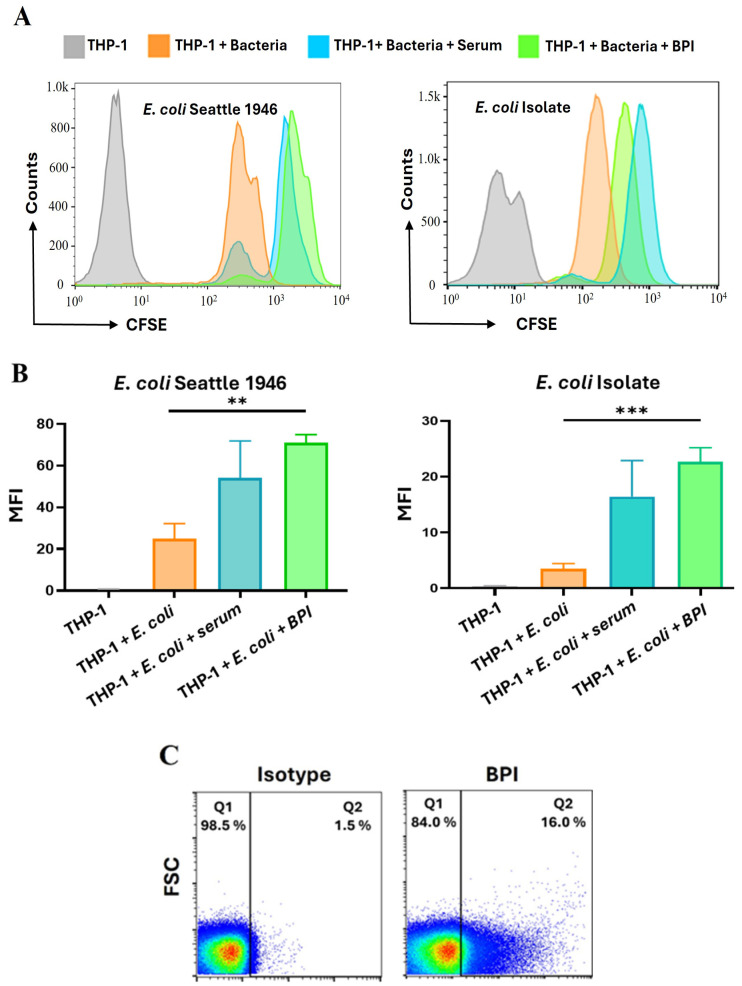
BPI used at colostrum concentrations favors the uptake of *E. coli* by THP-1 cells. THP-1 monocytes interacted with the *E. coli* Seattle 1946 strain and a clinical isolate stained with carboxyfluorescein succinimidyl ester (CFSE) and exposed to 1.695 μg/mL BPI or human serum before being incubated with THP-1 cells for 10 min. Finally, THP-1 cells were analyzed by flow cytometry. (**A**) **Left**: uptake of *E. coli* Seattle 1946 by THP-1 cells; **right**: uptake of *E. coli* clinical isolate by THP-1 cells. A representative histogram shows the binding of THP-1 cells (gray) to the bacteria in question, whether untreated (orange), pretreated with BPI (green), or in human serum (blue). (**B**) The graphs represent the mean ± standard deviation of five independent experiments showing the THP-1 cell binding to *E. coli* Seattle 1946 (**left**) and the clinical isolate (**right**). Statistical significance was assessed by a one-tailed ANOVA and is indicated as ** *p* < 0.01, and *** *p* < 0.001. (**C**) Flow cytometry analysis to evaluate the binding of BPI protein present in the protein fraction of human colostrum samples to *E. coli* Seattle 1946. A significant increase in BPI-positive bacteria is observed in the sample treated with Alexa Fluor 488-labeled anti-BPI compared to isotype control. Data represent the average of six independent experiments.

After determining the concentration of BPI (1.645 µg/mL) in colostrum capable of suppressing the growth of two different *E. coli* strains and recognizing that BPI can function as an opsonin [17], we investigated whether BPI promotes the uptake of *E. coli* by macrophages. The reference strain and the clinical isolate of *E. coli* (analyzed in the previous section) were labeled with the fluorescent tracer CFSE and incubated with either human serum or recombinant BPI at the average concentration found in colostrum. Subsequently, these bacteria were exposed to the THP-1 monocyte cell line. The results revealed that THP-1 cells exhibited significantly greater uptake of CFSE-labeled *E. coli* strains treated with recombinant BPI (70.95 ± 4.03 MFI for the *E. coli* Seattle 1945 strain and 22.7 ± 2.5 MFI for the clinical isolate) compared to untreated bacteria (3.49 ± 0.94 MFI). Moreover, BPI and human serum uptake effects were comparable (Figure 4A,B). Additionally, when the *E. coli* Seattle 1946 strain was treated with the soluble fraction of colostrum, we detected an interaction between BPI and *E. coli* using a monoclonal antibody specific to BPI (Figure 4C).

### 3.5. BPI at Colostrum Concentrations Inhibits E. coli-Induced Inflammation

**Figure 5:**
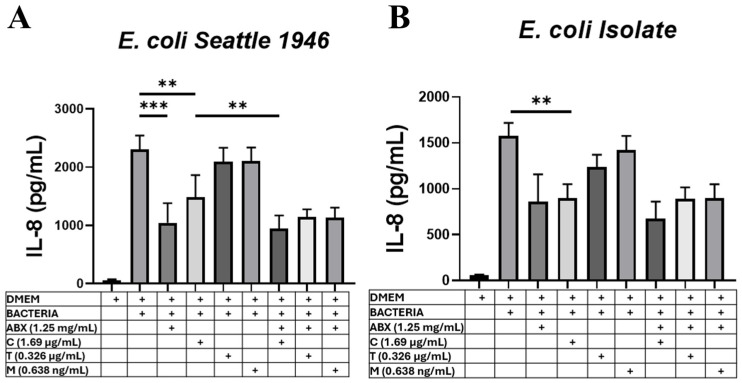
Determination of interleukin-8 (IL-8) release by HT 29 cells in the presence of BPI and *E. coli* strains. HT-29 cells were seeded and exposed to different conditions for two hours. Conditions included no bacteria present, inoculated with the Seattle 1946 strain of *E. coli* (**A**) or with the clinical isolate (**B**), inoculated with bacteria and the antibiotic amikacin, and inoculated with bacteria plus BPI at the mean concentrations found in each lactation phase (colostrum: 1.645 µg/mL; transition: 0.236 µg/mL; and mature milk: 0.638 ng/mL). In addition, the co-presence of the antibiotic and recombinant BPI was evaluated. Graphs represent the mean ± standard deviation of IL-8 release by HT-29 cells from six replicates, and statistical significance was determined by a one-tailed exhaustive ANOVA multiple comparison test, expressed as ** *p* < 0.01, and *** *p* < 0.001. DMEM (Dulbecco’s Modified Eagle Medium), C (colostrum), T (transition milk), M (mature milk), and ABX (antibiotic; amikacin).

Finally, concerning the anti-inflammatory capacity previously reported for BPI [18,19], we evaluated whether the average concentrations of BPI found in breast milk could inhibit inflammation in an intestinal epithelial cell infection model. To achieve our objective, the intestinal epithelial cell line HT-29 was infected with the *E. coli* strains used in the previous sections (Section 3.3 and Section 3.4) and treated with the average concentration of BPI found in each of the stages of breast milk or exposed to antibiotics or without treatment as controls. The supernatant of the THP-1 cells was collected to measure IL-8 secretion. Analysis of the results shows that only the average concentration of BPI found in colostrum can significantly inhibit IL-8 secretion by HT-29 cells infected with any of the *E. coli* strains used (Figure 5A,B). Interestingly, the inhibitory effect of BPI on IL-8 secretion at the average concentration in colostrum is similar to that of the antibiotic used (amikacin (50 mg/mL).

## 4. Discussion

The newborn faces several challenges at birth, including deficiencies in its immune system [20]. Breastfeeding is an effective form of protection, providing essential immune components such as soluble immunoglobulin IgA (sIgA) and antimicrobial proteins and peptides [21]. However, due to the complex composition of milk, the total content of antimicrobial molecules in breast milk is unknown. The results of this work demonstrate, for the first time, the presence of BPI in human breast milk. There are several proteomic analyses of human breast milk, but none of them have reported the presence of soluble BPI [22,23,24]. However, BPI inside the extracellular vesicles in human breast milk has already been reported [25]. The BPI concentrations we detected of 1.69 µg/mL in colostrum, 0.326 µg/mL in transitional milk, and 0.638 ng/mL for mature milk are lower than those reported for some antimicrobial molecules in breast milk. For example, lactoferrin is found in a concentration range of 9700–1140 µg/mL or lysozymes in 20.7–266 µg/mL. However, BPI is found at concentrations similar to or higher than other antimicrobial molecules in breast milk, such as HBD (human β-defensin)-1, with a concentration range of 0.0048 to 4.475 ng/L [26].

The next objective of our work was to identify the BPI-producing cells in breast milk. We found that CD11b^+^ leukocytes with high BPI expression are present in colostrum, while epithelial cells are present in a higher percentage; their BPI content is lower than in leukocytes. On the other hand, mature milk has a low rate of CD11b^+^ cells, which maintain a high expression of BPI and a high percentage of BPI^+^ epithelial cells. It is known that leukocytes [27,28], particularly neutrophils, have a high content of BPI [29,30]. It has also been reported that different populations of epithelial cells express BPI [31,32,33], but this is the first report that demonstrates the presence of BPI in epithelial cells present in breast milk. Previous work has also shown that during the colostrum stage, a considerable percentage of leukocytes declines in the mature milk stage, while epithelial cells remain at a high rate in both stages [14]. Interestingly, our results show a decrease in the number of BPI^+^ leukocytes and the concentration of soluble BPI in mature milk, suggesting that these cells could be responsible for the high concentrations of BPI in colostrum.

Subsequently, we found that the average concentration of BPI present in colostrum inhibits the growth of two different strains of *E. coli.* Similar results were obtained by Baricelli et al. [34], reporting that β2-defensin used at the concentration range present in colostrum (2.6–16.3 µg/mL) and mature milk (0.22–3.78 µg/mL) inhibits the growth of different bacterial strains isolated from diarrheal feces of children between 0 and 4 years of age. Another work demonstrates that mature milk contains both the transcript and the peptide of LL-37, and when the supernatant of denatured milk was supplemented with recombinant LL-37, they found that the minimum inhibitory concentration (MIC) to inhibit the growth of *S. aureus* and *E. coli* was 37 µM [35]. The N-terminal cationic end of BPI is responsible for antimicrobial activity because it binds to the negatively charged phosphate groups of LPSs in Gram-negative bacteria, displacing divalent cations, which alters the arrangement of LPS molecules, generating damage and rupture of the membrane [36]. Because the average concentration of recombinant BPI present in colostrum was the only one that inhibited the growth of *E. coli*, when using this concentration of BPI to supplement commercial formula milk, we found that it inhibited the development of the bacterial strains used in our study. Formula milk supplemented with lactoferrin has been associated with different benefits in reducing late-onset sepsis in preterm neonates [37]. In contrast, in infants, it reduces the frequency of lower respiratory tract diseases and higher hematocrit levels [38] and lower colonization by parasites of the genus *Giardia* [39]. Therefore, our results lead to further studies on the benefits of formula milk supplemented with BPI.

Our subsequent results show that when using the recombinant BPI protein at the average concentration in colostrum, there is a higher uptake of the two different strains of *E. coli* used in this work by THP-1 cells. It has been described that BPI-mediated opsonization/phagocytosis has a central role in the elimination of *P. aeruginosa* in vivo from a mechanism dependent on CD18 present in phagocytes [17]. Furthermore, by simulating the possible functions of BPI at the average concentration in colostrum, we demonstrated that BPI present in colostrum can bind to 1 × 10^6^ CFU of *E. coli* (16% of the bacteria). We consider that BPI in colostrum could have a higher binding if, in future assays, we used a lower number of CFUs and included the use of fresh colostrum because our assays were performed with this biological fluid stored and thawed, which, as previously described, affects the viability of the biological components of human milk [40]. Finally, we found that BPI treatment at average colostrum concentrations decreases IL-18 secretion by epithelial cells infected with *E. coli.* The anti-inflammatory function of BPI against Gram-negative bacteria is because its N-terminal region sequesters LPSs and prevents them from being correctly recognized by components of the TLR4 receptor complex [18]. In this context, it has been described that BPI inhibits TNF production in whole blood treated with Gram-negative bacteria (*E. coli*, *P. aeruginosa*, and *K. pneumoniae*) [19]. Likewise, overexpression of BPI in intestinal epithelial cells inhibits IL-8 secretion after infection with *S. typhimurium* [41].

On the other hand, the structural and physicochemical characteristics of BPI, which give it its antibacterial and anti-inflammatory function and its feasibility and safety, have led to the consideration of this molecule as a therapeutic agent for clinical use in research phases I, II, and III. Among the studies carried out, a reduction in mortality in children with meningococcal sepsis (clinical phases II and III) [11,42] and beneficial effects in patients with Crohn’s disease (clinical phase II) [43] stand out in both cases when treated with an N-terminal fragment of recombinant BPI (rBPI21).

The major weakness of this work lies in the need for assays showing the role of natural BPI in colostrum. For this reason, this limitation was addressed in the opsonization assays (Figure 4C). However, the contribution of BPI to the antimicrobial and anti-inflammatory activity of colostrum requires neutralization or removal of BPI, which is difficult due to the high concentrations of BPI at this stage in breast milk. In addition, there is a possibility that the functions of BPI in colostrum are redundant to other APPs. Therefore, we considered reproducing the individual activities of BPI in breast milk using recombinant BPI.

In conclusion, our results show that breast milk contains soluble BPI and leukocytes and epithelial cells that produce BPI. Furthermore, average concentrations of BPI in colostrum have antimicrobial, opsonizing, and anti-inflammatory activity against *E. coli.*

## Data Availability

The original contributions presented in this study are included in the article. Further inquiries can be directed to the corresponding author.
